# Computer Algorithms in the Search for Unrelated Stem Cell Donors

**DOI:** 10.1155/2012/175419

**Published:** 2012-11-01

**Authors:** David Steiner

**Affiliations:** Department of Cybernetics, Czech Technical University in Prague, Karlovo Náměstí 13, 121 35 Prague 2, Czech Republic

## Abstract

Hematopoietic stem cell transplantation (HSCT) is a medical procedure in the field of hematology and oncology, most often performed for patients with certain cancers of the blood or bone marrow. A lot of patients have no suitable HLA-matched donor within their family, so physicians must activate a “donor search process” by interacting with national and international donor registries who will search their databases for adult unrelated donors or cord blood units (CBU). Information and communication technologies play a key role in the donor search process in donor registries both nationally and internationaly. One of the major challenges for donor registry computer systems is the development of a reliable search algorithm. This work discusses the top-down design of such algorithms and current practice. Based on our experience with systems used by several stem cell donor registries, we highlight typical pitfalls in the implementation of an algorithm and underlying data structure.

## 1. Introduction

Hematopoietic stem cell transplantation (HSCT) [1] (commonly referred to as bone marrow transplantation) is a medical procedure in the field of hematology and oncology, most often performed for patients with certaincancersof thebloodorbone marrow. HSCT is the treatment of choice for people with hematopoietic malignancies, bone marrow failure, and certain types of cancer (e.g., lymphoma) which results in a compromised immune system. The most important factor in the successful outcome of HSCT is that the patient and donor are matched for the Human Leukocyte Antigens (HLA). The level of the matching required varies with the source of stem cells used for HSCT.

A lot of patients have no suitable HLA-matched donor within their family, so physicians must activate a “donor search process” by interacting with national and international donor registries who will search their databases for adult unrelated donors (AUD) or cord blood units (CBU). 

 Information and communication technologies play a key role in the donor search process in donor registries both nationally and internationaly. One of the major challenges for donor registry computer systems is the development of a reliable search algorithm. This work discusses the top-down design of such algorithms and current practice. Based on our experience with systems used by several stem cell donor registries, we will highlight typical pitfalls in the implementation of an algorithm and underlying data structure. 

## 2. Search Algorithm

The purpose of the donor search algorithm is to find and present a selected list of potential donors and/or CBUs, in which those most likely to be an optimal stem cell source for the patient are sorted to the top of the list [2]. Selection and sorting criteria are based on HLA compatibility and may also take into consideration secondary preference criteria, such as CMV antibody status, gender, and age.

Basic requirements for the search system used by stem cell donor registries are as follow.

- *Deterministic*: behavior that ensures the same results with the same input. This means, the algorithm has to reproduce exact decisions at every step.
- *Clear ranking order*: results.
- *Exhaustive*: all donors available for transplant in the source database should be included in the search algorithm. Exceptions must be clearly indicated to the end-user. For example, some algorithms exclude donors that are typed only at HLA-A and HLA-B.
- *Scalable*: the system should be able to handle databases of varying size and type.
- *Fast*: search algorithms are also used in user-interactive systems, so the results should be received in seconds.
- *Configurable*: search coordinator must be able to define patient-donor HLA match criteria and secondary preference criteria (CMV status, gender, and age).
- *Consistently matched*: The data presented should be uniformly matched as a set for a given instance of a patient search. Different primary algorithms or matching criteria shall not be used within a single patient search.

**Figure 1:**
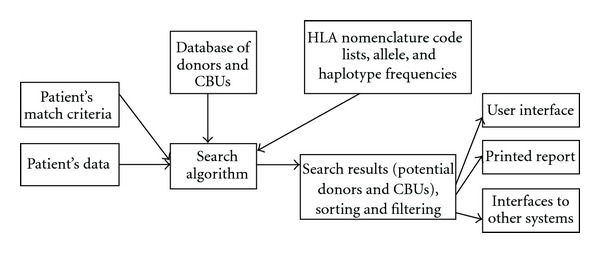
Basic concept of the donor search algorithm.

The search algorithm is usually implemented as the key component of the stem cell donor registry software system. It has several inputs and a single output (see Figure 1). The following input data are essential.

- Patient's data: HLA type (minimum HLA-A, HLA-B, and HLA-DRB1 typing).
- Patient's match criteria (position and number of allowable mismatches).
- Database of adult unrelated (AUD) and cord blood units (CBUs) (optional).
- HLA nomenclature code lists.
- Allele and haplotype frequencies (optional, depending on type of the algorithm).

The algorithm itself usually follows the following step.

- *Preprocessing*: fast preselection of donors based on predetermined internal indices.
- *Processing*: comparison of every (preselected) donor with the patient, calculation of match grades, matching probabilities, and filtering.
- *Postprocessing*: linking corresponding donor/CBU details.

The search output, which returns a sorted list of potential donors and CBUs can be presented either in the user interface, on a printed report, or transmitted to other systems (EMDIS). The presentation output may be calculated within the search engine software. For example, it is common practice to highlight patient-donor HLA mismatches as well as match grade and matching probability this may require additional data extraction from internal information calculated during the execution of the algorithm.

### 2.1. Patient's Data

Patient's HLA typing data must correspond to the valid HLA nomenclature and WMDA guidelines [3] and should be typed at the highest possible resolution, that is, at least intermediate resolution. Some algorithms may return unexpected search results, if low-resolution HLA typing data is provided.

**Example 1**

B∗35 : 76 has no mapping to “Unambiguous Serology” [4], but is mapped to “Possible Serology” B35 and B22. B22 is the broad HLA code with splits B54, B55, and B56. Therefore, a patient carrying B∗35: XX is a potential match with a donor carrying B56. Such a result is likely to be confusing for healthcare professionals. This problem would not appear if the patient was typed at higher resolution (the B∗35 : 76 allele is excluded). An alternative solution would be to apply an exceptions or filter by application of additional criteria, for example, matching probabilities with threshold (it is very unlikely that B∗35: XX will become B∗35 : 76).

### 2.2. Patient's Match Criteria

Some algorithms have hard-coded or fixed match criteria, but more sophisticated search algorithms allow users to define matching preferences for each individual search. EMDIS Matching Preferences [5] define the following criteria.

- Counting method for mismatches: count graft-versus-host (GvH) mismatches only or host-versus-graft (HvG) mismatches only.
- Maximum number of antigen/allele mismatches for adult donors.
- Maximum number of antigen/allele mismatches for CBUs.
- Maximum number of antigen/allele mismatches at loci A/A∗, B/B∗, Cw/C∗, DR/DRB1∗, DQ/DQB1∗.
- Additional sorting criteria like age of the donor, gender matching, and CMV matching.

### 2.3. Database of Donors and Cord Blood Units (CBUs)

Database of unrelated stem cell donors and CBUs should correspond to the following requirements [6].

- *Current*: the data used by the algorithm should be up to date.
- *Detailed*: the data presented should contain all relevant fields to the determination of match. The set of data elements should be consistent amongst the registry community.
- *Integrated*: the data presented should be considered as a set and should be available to the matching party as part of a singular search event.
- *Recognizable*: the data presented should uniquely reference individual sources using the identifier that is directly associated with the donor/CBU or would appear on any biological samples associated with the product.
- *Comprehensive*: the data presented should represent a consolidated view of the inventory. Uniform depth of access to all donors is needed.

Good implementation of the donor database is essential for acceptable performance of the search algorithm. Not all database structures of HLA applications are suitable as the data source for the algorithm.

Many small to middle size registry are colocated in a single centre with the HLA typing laboratory and there is a need for data integration of these two departments. It may seem that the registry system stores and manages the HLA typing results in the same way as the HLA laboratory information management system (LIMS), and some registries have implemented such data storage. It is a mistake to use these in search algorithms. The main differences between registry database and HLA LIMS database are as follow.

- The registry system needs fast access to the most current and comprehensive HLA typing results, which does not always mean the last test typing. This may be combination of multiple tests performed in the past by multiple typing techniques. The registry system always needs access to the full set of all loci that should be stored at one place, while the HLA lab system order includes only requested tests and loci, so HLA typing results of an individual may be spread in multiple typing orders.
- When the HLA lab supervisor approves the order results, it cannot be changed in the lab system. However, the registry system has to keep historical HLA typing results up to date according to the latest HLA nomenclature, so it needs to update them (deleted and renamed alleles, new HLA nomenclature).

Database of donors/CBUs can simply be organized in a single relational database table. Even this may be problematic. A logical database approach is to organize HLA code-lists in separated tables (multiple-allele codes, alleles, antigens, and their relations) and define master-detail relationship between donor data and HLA codes. These systems have been implemented in some registries. The storage of donor record is using only primary keys of HLA codes (as foreign keys). The disadvantage of the master-detail storage is that the retrieval of donor's HLA typing is inefficient. Often the solution for data retrieval in such a structure is cumbersome, because the database system has to join data (database natural join) from tens of tables or do tens of joins of the same table. The advantage is easy manipulation of the properties of HLA codes or even the renaming of HLA allele codes. But such operations are much less common, compared to data retrieval.

### 2.4. HLA Nomenclature Code Lists

In all cases, the algorithm has to recognize the description of HLA typing codes (e.g., multiple-allele codes) and relations between HLA codes, especially DNA to serology mapping. Some algorithms even use antigen recognition site matching, amino acid sequences, or nucleotide sequences. It is recommended that code lists and code attributes are downloaded from specialist reference websites [4, 7]. 

Donors have been typed by various different typing techniques and many of them are registered with HLA serological assignments. The database of donors could be preprocessed, so all interpretations and mapping of HLA codes could be saved in advance, but generally, the patient's HLA type is known only at the time of the search, so HLA nomenclature code lists are needed. Of some concern is that a minority of patients are still typed only by serologic typing techniques! This means that search algorithms must be capable of using these in the search process.

## 3. Preprocessing

Several variants of search algorithms are being used by stem cell donor registries. Selection of the algorithm is influenced by available resources, size of the donor database, availability of haplotype frequencies of the supported population(s), and so forth. We will discuss commonly used search algorithms.

### 3.1. Simple Preselection

The goal of the algorithm is to find potential donors for one patient. The phenotype of the patient is compared with all donors phenotypes in the donor registry database that are “available” for transplantation purposes (simple preselection).

For every donor D in the database

  Count Match Grade (patient P-donor D)

  If the Match Grade is acceptable, store

    data of donor D in the list of

    potential donors of patient P.

This kind of algorithm is usually used only for small to middle sized registries. Implementation enhancements can help to improve this situation. For example, increasing current capacities of server memories allows caching of all donors in the random access memory (RAM) of the server. The advantage of this algorithm is mainly in its simplicity and simple validation process. It also has very straightforward implementation of distributed or parallel computing. The drawback is the speed and memory limitation, especially where donor database is growing.

This algorithm could be extended to multiple patient searches that might be useful, for example, for EMDIS repeat searches [5], when search results from several thousands of donors have to be generated and compared with previous results. Again, the list of all patients could be cached in the server memory with one additional loop.

For every donor D in the database

  For every patient P in the database

    Count Match Grade (patient P-donor D)

    If the Match Grade is acceptable, store

      data of donor D in the list of

      potential donors of patient P.

### 3.2. Search Determinants

Databases from Registries and cord blood banks store the HLA types in many formats depending whether typing was by serology or by DNA-based methods. Registries must take these different assignments to create a match algorithm to search for a patient. This comparison is usually facilitated by the conversion of phenotypes to “search determinants” prior to development of matching algorithms [8]. 

The phenotype of the patient/donor is mapped to “Search Determinants” (SD) [9, 10]. The SD is a data record, based on serological antigens, corresponding to the original HLA phenotype. For example, it might be a group of six HLA, serologic-based assignments—three pairs for HLA-A, HLA-B, and HLA-DRB1 loci. There are also a number of issues with this approach, since some alleles have multiple or no serologic specificities. Therefore, an individual can have multiple SDs. SDs are used as an index to select the set of matching phenotypes. Then, more precise match grades are counted and the list of donors is filtered. 

The main application of SDs is the speeding up of the match process by using SDs as key values in conjunction with a database and a matching algorithm [11]. The main disadvantage is the need for regular checks and updates of SDs of all donors in the database; due to changes of donor data, HLA nomenclature updates and changes in the “DNA to serology” mapping. There are particular problems where there is no serological equivalent for a DNA allele.

### 3.3. DNA Matching Only

The National Marrow Donor Program (NMDP) in the United States has developed an algorithm [12] that does not use SDs for the initial matching step as this is done by directly comparing patient DNA type to donor DNA type. The algorithm is able to account for all serologic typing possibilities with the use of a special table called the “Serology to DNA Allele Table.” This table can be generated from the “rel_dna_ser.txt” and “rel_ser_ser.txt” files from hla.alleles.org [4].

## 4. Processing

**Figure 2:**
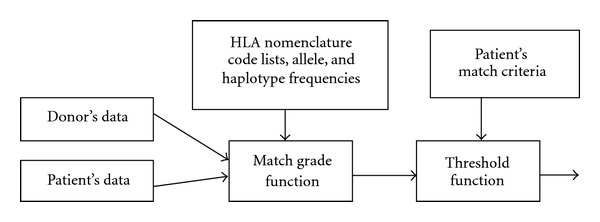
Match grade function.

The key element of the processing step of the algorithm is the “match grade function” that can compare data (HLA, ethnic group) of two individuals (usually patient and donor) and return their match grade and/or matching probabilities (see Figure 2). The threshold function then filters out donors that do not match patient's match criteria. 

Original versions of matching algorithms compared HLA typing only at HLA-A and HLA-B loci. DNA typing was not performed. Later generations added other loci, especially HLA-DRB1, but also HLA-C and HLA-DQB1. Today, some algorithms even use HLA-DRB3/4/5, HLA-DPB1, and other loci.

Earlier versions of matching algorithms also used only serological assignments; DNA typing either did not exist or was not taken into account. Later versions have converted DNA typing results into serological assignments or vice versa, so the algorithm has a uniform typing technique view on all donors. Current search algorithms use DNA typing results as much as possible and switch to serology comparisons only if DNA typing is not provided or if they want to refine DNA to serology mapping. 

The Information Technology (IT) Working Group of the World Marrow Donor Association (WMDA) has issued two key resources that describe the correct handling of HLA data and key patient-donor matching procedures:

- framework for the implementation of HLA matching programs in hematopoietic stem cell donor registries and cord blood banks [2]. This paper gives a bottom-up approach to the design of search algorithms: comparison of individual HLA codes, then HLA single-locus phenotypes, and eventually HLA multilocus phenotypes;
- fuidelines for use of HLA nomenclature and its validation in the data exchange among hematopoietic stem cell donor registries and cord blood banks [3].

A common mistake in the design of search algorithm is the violation of the rule 2.1 of the guidelines [3]: “laboratories must assign DNA nomenclature to results obtained using DNA-based methods and serologic nomenclature to results obtained using antibody reagents.” Some computer systems need to permanently store serology-derived results of DNA codes, usually because of simple DNA-serology matching. However, the mapping should be done automatically by the system and not by the user. Derived serology values must be clearly distinguished from real serology results obtained using antibody reagents. Where mapping has changed, the registry system has to know if stored serologic results should be updated or not. Moreover, some alleles are mapped to multiple serology equivalents and the system has to take this into account.

In addition to match grade, some information can be calculated. In these, the probability of HLA matching at the allele level based on local population haplotype frequencies in the underlying population can be calculated. Such prediction algorithm system has been developed and validated by the NMDP (HapLogic II) [13]. 

The latest, state-of-the-art versions of search algorithms (OptiMatch, HapLogic III) use these probability calculations to determine the rank order of HLA matches as the main searching and sorting criteria.

## 5. Postprocessing

At this stage, the system retrieves corresponding donor details of all selected donors that will be displayed in the search results. If the matching probabilities are not used as the main sorting criteria, the search system can apply them at this stage (ProMatch [14], Hap-E [15] and EasyMatch [16]). 

## 6. Probability Matching Algorithms

The search algorithms of the two largest registries in the world are based on the probability matching approach. 

Using a large number of high resolution HLA types the system can estimate the probability of other less well-typed donors being matched to the patient. The system is validated by retyping these donors to obtain high resolution types/haplotypes and thereby confirming that the calculation is accurate. The limitation of this is that it may be specific to an ethnic group.

### 6.1. OptiMatch

OptiMatch [17] is a matching program calculating, for each donor, the probability of allelic identity to the patient. The program was developed by the German registry ZKRD. The first version (October 2006) was based on 3 locus high resolution haplotype frequencies, while the current version (June 2008) is based on 5 locus high resolution haplotype frequencies. 

The web-based user interface lists potential donors with 7 probabilities: A∗ match, B∗ match, C∗ match, DRB1∗ match, DQB1∗ match, and overall probabilities of 10/10 match and 9/10 match.

### 6.2. HapLogic

The HapLogic program [13, 18] was developed by the NMDP registry. It works in a similar way to OptiMatch. First versions calculated probabilities of 6/6 allele matches, while the latest version III, introduced in November 2011, sorts donors based on probability of matching 10 alleles, using 5 locus high resolution haplotypes (like OptiMatch). HapLogic also uses 5 broad and 21 detailed race/ethnic groups. 

The web-based user interface shows a list of potential donors with several probabilities: A∗ match, B∗ match, C∗ match, DRB1∗ match, DQB1∗ match, and overall probabilities of 10/10 match, 9/10 match, 8/10 match, 8/8 match, 7/8 match, 6/8 match, and for cord blood units also 6/6 match, 5/6 match, and 4/6 match.

## 7. Implementation of the Probability Matching Algorithm

If the registry wants to implement probability matching algorithm, such as OptiMatch or HapLogic, it has to successfully complete the following three steps.

1. Design and implement the algorithm itself.
2. Estimate haplotype frequencies of the donor (and patient) populations—these 5 locus high resolution haplotype frequencies are usually estimated from a donor registry database.
3. Validate the search system—using retrospective data of historical searches. Usually, registry confirmatory typing requests (CTs) and their results are used.

There are two potential problems with the development of this approach: (1) unlike ZKRD and NMDP, other registries do not have sufficient donors to estimate 5 locus high resolution haplotype frequencies. Haplotype frequencies could be calculated, but their confidence is questionable. (2) Smaller registries also do not have enough high resolution HLA types (obtained at confirmatory typing, CT) for validation of the prediction algorithm. ZKRD used 9843 CTs in 2008 [17] and 22255 CTs in 2010 [19]. NMDP used about 60000 CTs (not published). These numbers are not achievable in smaller registries. 

In order to overcome these problems, the Prometheus system approximated the local population to the German (ZKRD) population, that is, by using ZKRD high resolution A∗-B∗-C∗-DRB1∗-DQB1∗ haplotype frequencies [14]. It also used high resolution HLA types from CT samples from multiple registries. 

## 8. Validation of the Search Algorithm

All implementations of the search algorithms need to be validated before being used. The WMDA Information Technology Working Group provides validation sets of patients and donors that are used for matching trials and comparison of results with expected outcomes [2, 20]. Algorithms that do not use simple preselection approach, but use more complex preselection, have to be validated for completeness. It is important not to miss any relevant donors in the preselection [2].

Validation of the processing phase, especially the match grade function, can be done by running several automated unit tests, addressing all kinds of matches and mismatches, exceptions, and rare cases. Interfaces to software source code classes, modules, or libraries are tested with a variety of input arguments to validate that the results that are returned are as expected [21]. 

The quality of prognostic matching algorithm and the population model used (allele and haplotype frequencies) also has to be validated. This is usually done by retrospective or prospective studies. Typically, all CTs performed by the registry that meet some criteria are used. These criteria are as the follows.

- Patient has been typed in high resolution.
- Donor was not typed in high resolution before the CT, but has been high resolution typed at the time of CT (or later).
- No discrepancy between a priori and final HLA type.

**Figure 3:**
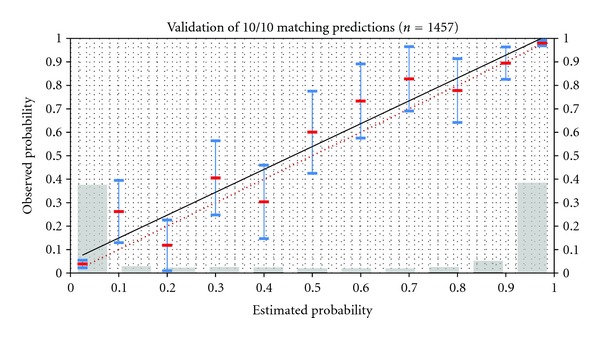
Prometheus probability matching algorithm (ProMatch): the graph shows the correlation of estimated 10/10 matching probabilities in 10% prediction intervals and corresponding observed probabilities. The population model is approximated by the German population. Blue bars show 95% confidence intervals of estimated probabilities. Grey bars show relative number of CTs in each prediction interval. Red-dotted line is the ideal correlation.

The review process retrospectively calculates the matching prognosis and compares the predicted and observed percentage of allele matches (see Figure 3). 

## 9. Conclusions

A reliable and efficient search algorithm is the key component of the unrelated stem cell donor registry computer system. An overview of search algorithms, their design, and implementation aspects have been described. Both combinatorial and probability matching algorithms have been presented.

A top-down design approach that first lists algorithm requirements, specifies input and output parameters, and then goes deeper into details was selected. The importance of validation prior to the implementation of a new matching algorithm has been emphasized.
